# Retinal vasculitis in *Toxocara canis* neuroretinitis

**DOI:** 10.1186/1869-5760-3-5

**Published:** 2013-01-03

**Authors:** Cagri G Besirli, Susan G Elner

**Affiliations:** 1 Department of Ophthalmology and Visual Sciences, University of Michigan Medical School, Ann Arbor, MI, 48105, USA; 2 Kellogg Eye Center, 1000 Wall Street, Ann Arbor, MI, 48105, USA

**Keywords:** *Toxocara canis*, Neuroretinitis, Vasculitis, Fluorescein angiography

## Abstract

**Background:**

The purpose of this case report is to describe clinical and angiographic findings of retinal vasculitis in acute *Toxocara canis* neuroretinitis associated with systemic infection.

**Findings:**

A 16-year-old male presented with a 1 week history of left eye pain, floaters, and decreased visual acuity. Ocular examination was consistent with neuroretinitis and retinal vasculitis. Fluorescein angiography demonstrated leakage of fluorescein from the optic nerve and the retinal veins. Clinical and laboratory evaluation were consistent with systemic *Toxocara canis* infection.

**Conclusions:**

Ocular *T. canis* may present with retinal vasculitis in young patients in the setting of acute systemic infection.

## Findings

### Introduction

*Toxocara canis (T. canis)* is a ubiquitous parasite found worldwide. *T. canis* can only complete its lifecycle in dogs, and humans are accidental hosts [1,2]. Ocular *T. canis* is typically seen in children with an average age of diagnosis of 7.5 years [2,3]. The majority of patients present with blurred vision, pain, photophobia, and floaters. *T. canis* may demonstrate a localized disciform macular detachment, multifocal granulomas with interconnecting tracks, peripheral retinal detachment, papillitis, peripheral retinal mass, pars plana mass, vitritis, endophthalmitis, or cataract. The most common causes for vision loss in the setting of ocular *T. canis* are dense vitritis, cystoid macular edema, and tractional retinal detachment. Although animal models of *T. canis* infection uniformly demonstrate retinal vasculitis [4], this finding has not been previously reported in human eyes. We report a patient who presented with acute onset vision loss in the setting of constitutional symptoms and a positive *T. canis* ELISA. Interestingly, this patient had clinical and angiographic findings consistent with retinal vasculitis. To our knowledge, this is the first report of retinal vasculitis in ocular *T. canis* infection.

### Case report

**Figure 1:**
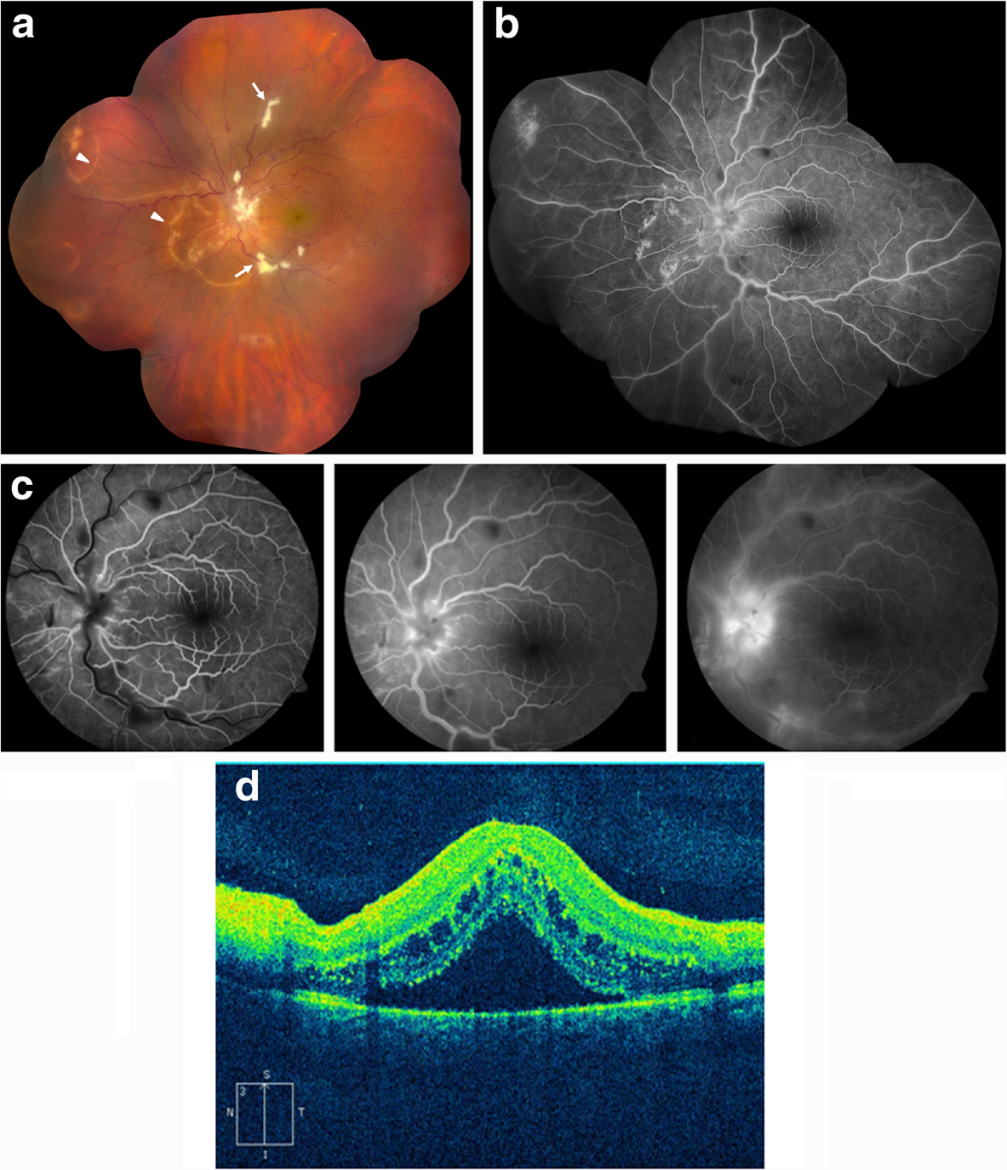
**Composite fundus photograph, fluorescein angiography and vertical OCT of the left eye.** (**a**) Composite fundus photograph of the left eye on the day of presentation demonstrating mild vitritis, optic nerve edema with overlying exudates, macular thickening, and multiple track-like chorioretinal scars (arrowheads). Retinal veins show phlebitis with areas of inflammatory exudates (arrows). (**b**) and (**c**) Fluorescein angiography of the left eye shows leakage of fluorescein from the optic nerve and the retinal veins. (**d**) Vertical OCT image of the left eye demonstrates subretinal and intraretinal fluid.

**Figure 2:**
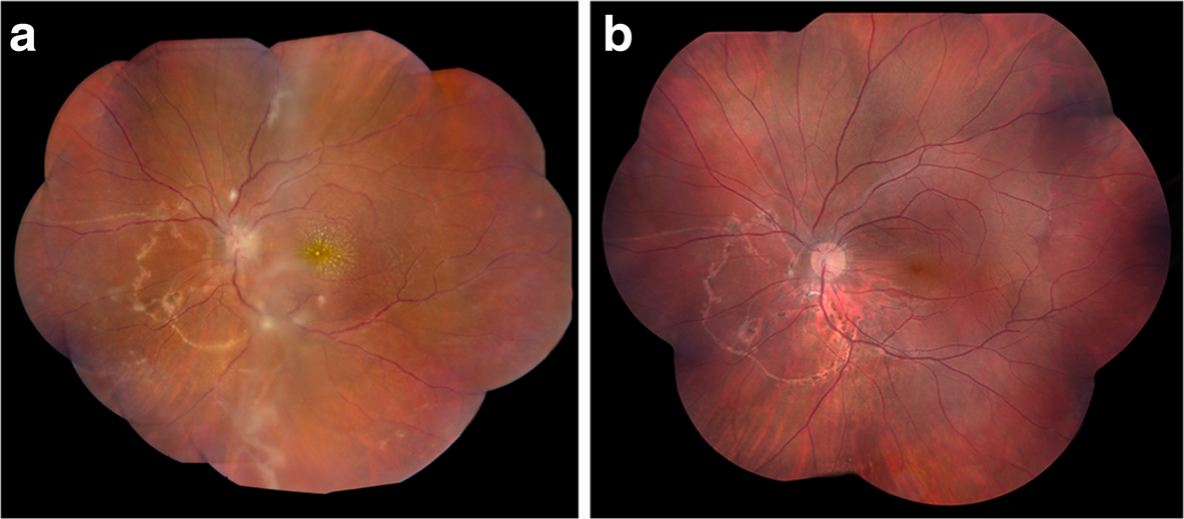
**Composite fundus photographs of the left eye 1 week and 17 months after initial presentation.** (**a**) Composite fundus photograph of the left eye 1 week after initial presentation shows the development of a macular star and resolving perivenular and peripapillary exudates. (**b**) Fundus examination showed post-infectious changes 17 months after presentation.

A 16-year-old male presented with a 1 week history of left eye pain, floaters, and decreased visual acuity. His review of symptoms was significant for headache, sore throat, and cough occurring 1 week prior to his visual symptoms. He did not have any known history of animal exposure and denied any recent direct contact with dogs. Visual acuity was 20/20 in the right eye and 4/200 in the left eye. The ophthalmic examination of the right eye was entirely normal. A relative afferent pupillary defect was present in the left eye. Anterior segment examination showed mild conjunctival hyperemia, fine keratic precipitates across the corneal endothelial surface, 3+ cells and flare in the anterior chamber, and 2+ anterior vitreous cells. Fundus examination showed mild posterior vitreous debris (Figure 1a). The optic nerve was swollen with overlying exudates and surrounding subretinal fluid. Macula was thickened with underlying subretinal fluid extending from the optic nerve. There were multiple, track-like chorioretinal scars around the nasal portion of the retina, near the optic disk, and extending into the periphery (Figure 1a). Retinal veins showed mild phlebitis with areas of cotton wool spot-like exudates along the temporal vascular arcades. Fluorescein angiography of the left eye showed leakage of fluorescein from the optic nerve and the retinal veins (Figure 1b, c). Optical coherence tomography (OCT) of the left eye showed subretinal and intraretinal fluid (Figure 1d). The patient was started on topical steroid and cycloplegic treatment, and laboratory work-up including PPD with control, CBC, angiotensin converting enzyme, FTA-ABS, c-ANCA, HLA-B5, HLA-B27, *Bartonella* panel, *T. canis* ELISA, and toxoplasmosis IgG and IgM was initiated. A chest X-ray was ordered. One week later, visual acuity measured 20/200 on the left with improving anterior chamber inflammation. Funduscopic examination showed decreased subretinal fluid, development of a macular star, and resolving perivenular and peripapillary exudates (Figure 2a). The laboratory work-up returned positive for *T. canis* ELISA and elevated eosinophil count at 8.2%. The rest of the laboratory work-up and chest X-ray were within normal limits. The patient's positive *T. canis* ELISA, eosinophilia, and systemic symptoms strongly indicated the diagnosis of systemic *T. canis* infection, and after consultation with the infectious disease service, he was treated with albendazole and oral prednisone. Two weeks later, the visual acuity remained at 20/200, and fundus examination showed improving macular star, optic nerve edema, and retinal exudates. Visual acuity improved to 20/80 17 months after his initial presentation (Figure 2b).

### Discussion

Ocular manifestations of *T. canis* vary greatly and may include disciform macular detachment, multifocal granulomas, retinal tracks, peripheral retinal detachment, papillitis, peripheral retinal mass, pars plana mass, vitritis, endophthalmitis, and cataract. However, to our knowledge, retinal vasculitis has not been previously described in patients with ocular *Toxocariasis*. Our patient presented with clinical and angiographic findings of retinal vasculitis in the setting of *T. canis* neuroretinitis and evidence of concurrent systemic *T. canis* infection. His examination was significant for sheathing of the retinal veins and cotton wool spot-like exudates. Fluorescein angiography demonstrated dye leakage from retinal vessels with a venous predominance, consistent with the clinical findings.

Vasculitis is a well-documented finding in many organs in patients affected by systemic *T. canis* infection [5]. In addition, retinal vasculitis is one of the common findings in animal models of ocular *T. canis* infection [4]. Most patients with ocular *T. canis* are not seen in the acute phase of systemic infection. This may partly explain why retinal vasculitis is an uncommon finding in human ocular infections with *T. canis* and has not been reported previously. In our patient, the presence of constitutional symptoms, positive *T. canis* ELISA, and increased eosinophil count on blood analysis indicated concurrent systemic and ocular *T. canis* infection.

### Conclusion

Although uncommon, *T. canis* infection needs to be considered in the differential diagnosis of neuroretinitis and retinal vasculitis in young patients. Evaluation for constitutional symptoms of *T. canis* infection as well as laboratory work-up including *T. canis* ELISA and complete blood count may assist with diagnosis. Treatment with anthelmintic agents and systemic steroids may hasten recovery of ocular symptoms and funduscopic findings, though permanent posterior segment changes are common secondary to infectious and inflammatory factors. Visual outcome may be limited due to irreversible retinal damage despite the initiation of anthelmintics and anti-inflammatory agents.

### Consent

Informed consent was obtained from the mother of patient on his behalf for publication of this report and any accompanying images.

## Competing interests

The authors declare that they have no competing interests.

## Authors’ contributions

CB and SE conceived of the study, participated in its design and coordination, and helped to draft the manuscript. All authors read and approved the final manuscript.
